# Chain mediation of rumination and negative coping styles in the relationship between fertility stress and psychological distress among Chinese infertile women: a cross-sectional study

**DOI:** 10.3389/fpsyg.2026.1784271

**Published:** 2026-04-13

**Authors:** Siyan Liu, Dongming Qu, Junru Li, Hongmei Wu, Wei Wang

**Affiliations:** 1 Nursing Department, The Fifth Affiliated Hospital of Harbin Medical University, Daqing, China; 2 Daqing People’s Hospital, Daqing, China; 3 Department of Reproductive Medicine, The First Affiliated Hospital of Harbin Medical University, Harbin, China; 4 College of Nursing, Harbin Medical University-Daqing Campus, Daqing, China; 5 Laboratory of Basic Research and Health Management on Chronic Diseases in Heilongjiang Province, Daqing, China; 6 Obstetrics Department, The Fifth Affiliated Hospital of Harbin Medical University, Daqing, China

**Keywords:** chain mediation, Chinese women, cognitive vulnerability, fertility stress, infertility, negative coping styles, psychological distress, rumination

## Abstract

**Background:**

Infertility affects approximately 48.5 million couples worldwide, with infertile women experiencing significantly higher rates of psychological distress (27–40%) compared to the general population. While fertility stress has been identified as a primary contributor to psychological distress, the underlying mechanisms remain poorly understood.

**Objective:**

This cross-sectional study examined the mediating roles of rumination and negative coping styles in the relationship between fertility stress and psychological distress among Chinese infertile women. We hypothesized that fertility stress would influence psychological distress through three indirect pathways: (1) rumination alone, (2) negative coping styles alone, and (3) a sequential chain from rumination to negative coping styles.

**Methods:**

A total of 608 infertile women (mean age 33.5 ± 4.8 years) were recruited from three tertiary hospitals in Heilongjiang Province, China. Participants completed standardized measures including the Fertility Problem Inventory (FPI), Event-Related Rumination Inventory (ERRI), Trait Coping Style Questionnaire (TCSQ), and the Chinese version of the Infertility Distress Scale (IDS). Chain mediation analysis was conducted using the PROCESS macro (Model 6) with 5,000 bootstrap samples.

**Results:**

Psychological distress demonstrated strong positive correlations with fertility stress (*r* = 0.43, *p* < 0.01), rumination (*r* = 0.53, *p* < 0.01), and negative coping styles (*r* = 0.49, *p* < 0.01). Chain mediation analysis revealed that the total effect of fertility stress on psychological distress was *b* = 0.20 (95% CI [0.16, 0.23]). The direct effect accounted for 45% of this relationship, while indirect effects accounted for 55%. Three significant mediation pathways were identified: through rumination alone (30% of total effect), through negative coping styles alone (20% of total effect), and through the sequential chain from rumination to negative coping styles (5% of total effect).

**Conclusion:**

Fertility stress influences psychological distress in infertile women through direct and indirect pathways. A theoretically-derived sequential chain model suggests a potential vulnerability mechanism, though the cross-sectional design does not permit verification of causal temporal ordering. Longitudinal research is needed to confirm the hypothesized sequence. These findings suggest rumination may represent a key intervention target. However, given the cross-sectional design, further longitudinal and intervention research is needed to establish whether rumination-focused approaches effectively reduce psychological distress. Potential clinical implications should be considered preliminary and warrant testing through randomized controlled trials.

## Introduction

Infertility, defined by the World Health Organization as the inability to achieve pregnancy after 12 months of regular unprotected sexual intercourse (Inhorn, 2003), affects approximately 48.5 million couples globally. In China, the prevalence of infertility has risen to 12–18%, with an estimated 50 million individuals affected (Xie et al., 2022). This condition not only poses biological challenges but also imposes substantial psychological burdens, particularly on women, who often bear disproportionate social and familial pressures related to reproduction (Samadaee-Gelehkolaee et al., 2015).

### Psychological distress: the sixth vital sign in infertility

Psychological distress, characterized by unpleasant emotional experiences arising from psychological, social, and spiritual challenges, has been recognized as the “sixth vital sign” in healthcare, alongside temperature, respiration, blood pressure, pulse, and pain (Thomas et al., 2023). Research indicates that 27–40% of infertile women experience clinically significant psychological distress, including anxiety and depression—rates substantially higher than those observed in the general female population (Chernoff et al., 2021). Unlike transient emotional reactions, psychological distress among infertile women is chronic and multifaceted, stemming from fertility concerns (Tan et al., 2008), identity threats, marital strains, societal stigma, and treatment uncertainties (Foti et al., 2023).

In Chinese cultural contexts, the burden of infertility is particularly pronounced. Confucian values traditionally emphasize family lineage and filial piety through offspring, creating social expectations around reproductive success (Wang and Wu, 2026). Infertile Chinese women often face intense social scrutiny from extended family members, relatives, and community networks. These culturally-specific social pressures may create distinct psychological stressors that warrant culturally-informed research. Understanding the unique mechanisms through which infertility-related stressors translate into psychological distress in this population is therefore important for both theoretical understanding and clinical practice.

### Fertility stress as a primary stressor

Fertility-related stress encompasses the multidimensional pressures experienced by infertile individuals, including concerns about conception, invasive medical procedures, financial burdens, sexual difficulties, and social stigmatization (Bjornsdottir et al., 2023). Previous research has established that fertility stress positively predicts psychological distress in infertile women (Kamboj et al., 2023). However, empirical research indicates substantial individual differences in psychological responses to fertility stress (Kamboj et al., 2023). According to the Transactional Theory of Stress and Coping (Spataru et al., 2024), cognitive processes and behavioral responses mediate between stressors and psychological outcomes. Some women develop severe psychological distress in response to fertility stress, while others demonstrate resilience despite comparable stress levels, a phenomenon documented in resilience research among infertile populations (Darolia and Ghosh, 2022). The present study examines whether rumination and negative coping styles explain why some women develop severe psychological distress while others demonstrate resilience despite comparable fertility stress levels. This approach allows us to identify the specific mechanisms underlying heterogeneous responses to infertility.

### Rumination: a cognitive vulnerability factor

According to Response Styles Theory (Nolen-Hoeksema, 1991), rumination defined as repetitive, passive focus on distress symptoms and their causes and consequences represents a negative cognitive response style that intensifies and prolongs negative emotional states. Rumination directs attentional resources toward negative information, impairs problem-solving abilities, and maintains depressive symptoms (Lyubomirsky et al., 2015).

In the context of infertility, women often engage in intrusive rumination about their reproductive failure, the uncontrollability of their condition, and the perceived gap between their expectations and reality (Tasuji et al., 2020). Previous research has demonstrated that rumination mediates the relationship between negative life events and depression in various populations (Roberts et al., 2013; Li et al., 2024). However, its specific role in linking fertility stress to psychological distress among infertile women remains under explored. No study has systematically examined whether rumination serves as a mediator in the fertility stress-psychological distress relationship among Chinese infertile women.

### Coping styles: a behavioral response mechanism

Coping styles refer to cognitive and behavioral styles individuals employ to manage stressful situations (Yin et al., 2022). The stress process model posits that coping styles play a crucial role in determining mental health outcomes following exposure to stressors (Dai et al., 2023). Coping styles are broadly categorized into positive (problem-focused, seeking support, cognitive reframing) and negative (avoidance, denial, emotional venting) styles. In the context of infertility-related stress, research suggests that negative coping styles are specifically associated with increased psychological distress, whereas the absence of positive coping styles appears less central to the distress pathway. Avoidance, denial, and emotional suppression may provide temporary relief but ultimately prevent adaptive adjustment and exacerbate distress over time (Khalid and Dawood, 2020). Our focus on negative coping styles rather than positive coping styles is theoretically justified because the stress-to-distress pathway in infertile women is driven primarily by the active engagement in negative behaviors, not by the lack of adaptive styles. Therefore, we examined whether negative coping styles mediates the fertility stress-psychological distress relationship in Chinese infertile women.

### The sequential chain: from rumination to negative coping styles

Beyond examining rumination and coping styles as independent mediators, theoretical models suggest these factors may operate sequentially. According to Response Styles Theory, rumination consumes cognitive capacity by directing attention toward emotional states rather than problem-solving (Dell'Osso et al., 2021). This cognitive resource depletion subsequently reduces individuals’ ability to employ effective coping styles, promoting reliance on negative coping styles behaviors. The Stress-Cognitive Vulnerability Interaction Model formalizes this mechanism: exposure to negative life events triggers rumination, which impairs cognitive resources and promotes adoption of negative coping styles (Hankin and Abramson, 2001). Empirical evidence supports this sequential pathway: with research showing rumination predicts greater use of negative coping styles and worse mental health outcomes (McCarrick et al., 2023).

In adolescent populations, research has demonstrated that rumination and negative coping styles exert a chain mediating effect between negative life events and depression (Guo et al., 2011). Despite theoretical support and evidence from other populations, no study has tested whether rumination and negative coping styles sequentially mediate the relationship between fertility stress and psychological distress in infertile women. Understanding this chain mechanism could reveal critical intervention points suggesting that early cognitive interventions targeting rumination may prevent the downstream adoption of negative coping styles.

### The present study

This study addresses the aforementioned research gaps by examining a comprehensive mediation model in which rumination and negative coping styles serve as both independent and sequential mediators linking fertility stress to psychological distress among Chinese infertile women. Based on Response Styles Theory and the Stress-Cognitive Vulnerability Interaction Model, we tested the following hypotheses:

*Hypothesis 1 (H1)*: Fertility stress is positively associated with psychological distress.

*Hypothesis 2 (H2)*: Rumination independently mediates the relationship between fertility stress and psychological distress.

*Hypothesis 3 (H3)*: Negative coping styles independently mediates the relationship between fertility stress and psychological distress.

*Hypothesis 4 (H4)*: Rumination and negative coping styles sequentially mediate the relationship between fertility stress and psychological distress. This ordering reflects Response Styles Theory’s premise that rumination depletes cognitive resources, thereby promoting reliance on negative coping styles (fertility stress → rumination → negative coping styles → psychological distress). We focused our mediation hypotheses on negative coping styles rather than positive coping styles. Although the TCSQ measures both positive and negative coping dimensions, theory and prior research in the infertility context suggest that psychological distress is driven by active engagement innegative behaviors (negative coping styles) rather than by deficits in adaptive behaviors (positive coping styles). Therefore, we tested only negative coping styles as a mediator.

By testing these hypotheses, this study contributes to theoretical understanding of psychological distress development in infertile women and provides evidence-based guidance for designing staged interventions that target specific cognitive and behavioral mechanisms.

## Methods

### Study design and setting

This cross-sectional study was conducted between October 2022 and August 2023 in three tertiary-level hospitals in Heilongjiang Province, China. These hospitals are major reproductive medicine centers serving both urban and rural populations in northeastern China. The study protocol was approved by the Research Ethics Committee of Daqing Campus, Harbin Medical University (Reference No. HMUDQ20240323002) and adhered to the principles of the Declaration of Helsinki.

### Participants

#### Inclusion criteria

Participants were eligible if they: (1) met the World Health Organization diagnostic criteria for infertility (i.e., women of reproductive age who had regular unprotected sexual intercourse for at least 12 months without achieving pregnancy); (2) were aged 20–45 years; (3) had an educational level of primary school or above; (4) had no diagnosed cognitive or communication disorders; (5) were able to read and comprehend Mandarin Chinese; and (6) provided voluntary informed consent.

#### Exclusion criteria

Participants were excluded if they: (1) had serious concurrent physical illnesses (e.g., cancer, severe cardiovascular disease) or diagnosed psychiatric disorders (e.g., major depressive disorder, anxiety disorders) that existed prior to infertility diagnosis; (2) were currently receiving psychiatric treatment or psychotropic medications; (3) had pregnancy complications or were currently pregnant; or (4) withdrew from the study before completing all questionnaires.

### Sample size determination

*A priori* power analysis was conducted using G*Power 3.1.9.7 software. Based on previous mediation studies examining psychological factors in infertile populations, we anticipated a medium effect size (*f*^2^ = 0.15) (Correll et al., 2020). With *α* set at 0.05, desired power at 0.80, and accounting for four predictors in the regression model, the minimum required sample size was calculated as 85 participants. However, for mediation analysis using bootstrap procedures, Hayes (2018) recommends a minimum sample size of 500 to ensure stable estimates of indirect effects. Therefore, we aimed to recruit at least 550 participants, with an anticipated 10% attrition rate.

### Sampling and recruitment procedure

Convenience sampling was employed to recruit participants from the outpatient clinics and inpatient wards of the reproductive medicine departments in the three participating hospitals. Trained research assistants (graduate students in nursing and psychology) approached eligible women during their clinic visits or hospital stays. The research assistants explained the study purpose, assured confidentiality, and obtained written informed consent from those who agreed to participate.

Data collection was conducted through face-to-face interviews in private consultation rooms to ensure confidentiality. Participants completed paper-based questionnaires independently, with research assistants available to clarify questions if needed. Each assessment session lasted approximately 30–40 min. To ensure data quality, research assistants reviewed completed questionnaires immediately for missing items or inconsistent responses and asked participants to clarify when necessary.

A total of 640 women were initially recruited. After excluding 32 questionnaires due to incomplete responses (>20% missing data, *n* = 24) or clearly patterned responses (e.g., selecting the same response option for all items, *n* = 8), 608 valid questionnaires were retained, yielding an effective response rate of 95%.

### Data collection instruments

#### Demographic and clinical variables

A self-designed questionnaire collected demographic information including age, education, employment status, place of accommodation (urban/country), monthly income, and whether the participant or her husband is the only child. Clinical variables included type of diagnosis (primary/secondary), duration of infertility, history of abortion, previous treatments, treatments with *in vitro* fertilization (IVF) (frequency), and whether medical insurance is available to cover the cost of treatment.

#### Fertility stress: fertility problem inventory (FPI)

Fertility stress was assessed using the Chinese version of the Fertility Problem Inventory (FPI), originally developed by Newton et al. (1999) and translated into Chinese by Peng et al. (2011). The FPI comprises 46 items across five subscales: social concern (10 items), sexual concern (8 items), relationship concern (10 items), rejection of childfree lifestyle (8 items), and need for parenthood (10 items). Items are rated on a 6-point Likert scale ranging from 1 (strongly disagree) to 6 (strongly agree), with total scores ranging from 46 to 276. Although the FPI and Infertility Distress Scale (IDS) share some measurement domains (e.g., social concerns), they represent conceptually distinct constructs. The FPI measures fertility related stressors (specific pressures), while the IDS measures psychological distress outcomes (emotional consequences). This distinction aligns with stress-outcome frameworks where stressors predict outcomes through mediating mechanisms. Higher scores indicate greater fertility-related stress. The Chinese version has demonstrated good reliability and validity in infertile Chinese women (Peng et al., 2011). In the current study, Cronbach’s *α* was 0.98 for the total scale, with subscale alphas ranging from 0.88 to 0.94.

#### Rumination: event-related rumination inventory (ERRI)

Rumination was measured using the Chinese version of the Event-Related Rumination Inventory (ERRI), developed by Cann et al. (2011) and translated by Dong et al. (2013). The ERRI contains 20 items assessing two dimensions: intrusive rumination (10 items, reflecting unwanted, repetitive thoughts about the stressful event) and deliberate rumination (10 items, reflecting purposeful attempts to make sense of the experience). Items are rated on a 4-point scale from 0 (not at all) to 3 (often), with total scores ranging from 0 to 60. Higher scores indicate greater levels of rumination. The Chinese version has shown adequate psychometric properties in Chinese trauma populations (Dong et al., 2013). In this study, Cronbach’s *α* was 0.95 for the total scale, 0.92 for intrusive rumination, and 0.91 for deliberate rumination. In this study, the stressful event was operationalized as participants’ overall infertility experience (diagnosis, treatment, and psychosocial challenges). Research assistants clarified this reference point to ensure standardized interpretation.

#### Coping styles: trait coping style questionnaire (TCSQ)

Coping styles were assessed using the Trait Coping Style Questionnaire (TCSQ) developed by Jiang and colleagues specifically for Chinese populations. The TCSQ consists of 20 items measuring two dimensions: positive coping styles (10 items, e.g., problem-solving, seeking help, positive reappraisal) and negative coping styles (10 items, e.g., avoidance, denial, venting). Items are rated on a 5-point Likert scale from 1 (definitely not) to 5 (definitely yes), with scores for each subscale ranging from 10 to 50. Higher scores indicate greater use of that coping style. The TCSQ has been widely used in Chinese medical populations and demonstrates good reliability (Yang et al., 2020). In the present study, Cronbach’s *α* was 0.82 for positive coping and 0.84 for negative coping styles. Although both subscales were assessed, only negative coping styles was included in the mediation analysis, consistent with theoretical predictions that negative coping styles (rather than positive coping styles) mediates the fertility stress-distress relationship in this population.

#### Psychological distress

The Chinese version of the Infertility Distress Scale (IDS) was used to measure psychological distress among infertile women (Liu et al., 2025). This scale comprises 23 items, with items 5, 13, 14, and 21 scored in the opposite direction. The scores range from 1 (never feel) to 4 (always feel). This scale encompasses four dimensions, namely, social distress (9 items), marital distress (7 items), subjective experience (4 items), and treatment distress (3 items). The results of the validation indicated the good model fit, with a Cronbach’s *α* coefficient being 0.92.

#### Data analysis

All statistical analyses were conducted using IBM SPSS Statistics version 22.0 (IBM Corp., Armonk, NY, USA) and the PROCESS macro version 3.5 developed by Andrew Hayes. Since all measures were self-reported and collected at a single time point, potential common method bias (CMB) represents a methodological concern that warrants careful consideration. We assessed CMB using Harman’s single-factor test, wherein all items from the four main scales were entered into an unrotated exploratory factor analysis. If a single factor accounts for more than 40% of the total variance, common method bias is considered problematic. However, as documented in the methodology literature (Wei et al., 2022), this traditional approach has well-recognized limitations in detecting latent method effects and should not be interpreted as definitive exclusion of CMB. Therefore, we adopted a cautious interpretive stance regarding potential influences of method variance on observed relationships. Demographic and clinical characteristics were described using frequencies and percentages for categorical variables and means with standard deviations for continuous variables. Missing data were minimal (<5% for any variable). Given the low missingness rate, listwise deletion was applied. Pearson correlation coefficients were calculated to examine bivariate relationships among fertility stress, rumination, positive coping styles, negative coping styles, and psychological distress. Prior to conducting the regression analysis for the mediation model, multicollinearity was assessed using tolerance and variance inflation factor (VIF). A VIF value of 5 was set as the critical threshold for serious multicollinearity. Chain mediation analysis was conducted using the PROCESS macro Model 6 to tests chain mediation. The directional ordering (fertility stress → rumination → negative coping styles → psychological distress) is theoretically grounded in Response Styles Theory: rumination depletes cognitive resources, thereby increasing vulnerability to negative coping styles. The independent variable was fertility stress, the dependent variable was psychological distress, and the mediators were rumination (M1) and negative coping styles (M2). Unless otherwise noted, all regression coefficients reported in the Results section are unstandardized (*b*). Statistical significance was set at *α* = 0.05 (two-tailed) for all tests.

## Results

### Common method bias test

Harman’s single-factor test was conducted to assess potential common method bias. Unrotated exploratory factor analysis revealed that the first factor accounted for 27.60% of the total variance, well below the 40% critical threshold.

### Participant characteristics

Demographic and clinical characteristics of the 608 participants are presented in Table 1. The mean age was 33.5 years (SD = 4.8), with the majority (78.29%) aged 30–40 years, representing the peak reproductive years. Over two-fifths (42.43%) held a bachelor’s degree or higher, and the vast majority (88.32%) resided in urban areas. Monthly household income was distributed as follows: ≤5,000 RMB (34.54%), 5,000–10,000 RMB (44.08%), and >10,000 RMB (21.38%).

**Table 1:** Participant characteristics (*N* = 608).

Variable	Group	*N*	%
Age (years)	<30	61	10.03
	30 ~ 40	476	78.29
	>40	71	11.68
Education	Junior high school or below	114	18.75
	High school	61	10.03
	College or associate degree	175	28.78
	University and above	258	42.43
Employment status	Unemployed	122	20.07
	Worker	36	5.92
	Farmer	36	5.92
	Science, education, culture and health personnel	99	16.28
	Individual business	74	12.17
	Other	241	39.64
Place of accommodation	Urban	537	88.32
	Country	71	11.68
The only child	Yes	296	48.68
	No	312	51.32
Husband is the only child	Yes	337	55.43
	No	271	44.57
Monthly income (RMB)	0 ~ 5,000	210	34.54
	5,000 ~ 10,000	268	44.08
	10,000~	130	21.38
History of abortion	0	482	79.28
	1	107	17.60
	≥2	19	3.13
Duration of infertility (years)	<2	82	13.49
	2 ~ 5	167	27.47
	>5	359	59.05
Whether medical insurance is available to cover the cost of treatment	Yes	155	25.49
	No	453	74.51
Type of diagnosis	Primary infertility	259	42.60
	Secondary infertility	349	57.40
Previous treatments	Yes	381	62.66
	No	227	37.34
Treatments with *in vitro* fertilization (IVF) (frequency)	0	227	37.34
	1	206	33.88
	≥2	175	28.78

Given the sample size, *N* = 608, this table presents the classification details and proportion information for 13 variables.

Regarding clinical characteristics, secondary infertility (57.40%) was more prevalent than primary infertility (42.60%). The majority (59.05%) had experienced infertility for more than 5 years, while 27.47% had been infertile for 2–5 years, and only 13.49% for less than 2 years. Most participants (79.28%) had no history of abortion. Among the sample, 62.66% had undergone previous assisted reproductive technology (ART) treatments, with IVF cycle distribution as follows: no IVF cycles (37.34%), one cycle (33.88%), and two or more cycles (28.78%). Notably, only 25.49% had medical insurance coverage for infertility treatment, suggesting substantial out-of-pocket financial burdens for most participants.

### Univariate analysis of psychological distress

Comparative analyses of total psychological distress scores among infertile patients with different participant characteristics showed that there were statistically significant differences in total psychological distress scores by age, employment status, and treatments with *in vitro* fertilization (IVF) (*p* < 0.05). As shown in Table 2.

**Table 2:** Comparison of total psychological distress scores by participant characteristics.

Variable	Group	Mean ± SD	*F*	*t*	*p*
Age (years)	<30	58.48 ± 24.61	3.91	–	<0.05
	30 ~ 40	59.53 ± 20.10			
	>40	52.13 ± 21.91			
Education	Junior high school or below	57.34 ± 19.56	1.37	–	>0.05
	High school	58.39 ± 22.46			
	College or associate degree	61.22 ± 21.93			
	University and above	58.56 ± 20.90			
Employment status	Unemployed	57.25 ± 22.22	3.89	–	<0.05
	Worker	47.39 ± 9.49			
	Farmer	65.36 ± 23.81			
	Science, education, culture and health personnel	62.83 ± 21.99			
	Individual business	57.19 ± 20.80			
	Other	58.55 ± 19.93			
Place of accommodation	Urban	58.71 ± 21.01	–	0.48	>0.05
	Country	57.44 ± 20.22			
The only child	Yes	58.61 ± 21.33	–	0.06	>0.05
	No	58.51 ± 20.53			
Husband is the only child	Yes	58.90 ± 19.73	–	0.45	>0.05
	No	58.14 ± 22.32			
Monthly income (RMB)	0 ~ 5,000	61.11 ± 20.90	2.99	–	>0.05
	5,000 ~ 10,000	57.10 ± 20.88			
	10,000~	55.60 ± 20.64			
History of abortion	0	58.94 ± 20.12	0.78	–	>0.05
	1	57.80 ± 24.44			
	≥2	53.16 ± 19.08			
Duration of infertility(years)	<2	62.44 ± 27.65	2.51	–	>0.05
	2 ~ 5	59.71 ± 21.98			
	>5	57.14 ± 18.39			
Whether medical insurance is available to cover the cost of treatment	Yes	57.21 ± 21.91	–	0.93	>0.05
	No	59.02 ± 20.56			
Type of diagnosis	Primary infertility	60.17 ± 20.86	–	1.64	>0.05
	Secondary infertility	57.36 ± 20.89			
Previous treatments	Yes	59.06 ± 19.69	–	0.73	>0.05
	No	57.73 ± 22.83			
Treatments with *in vitro* fertilization (IVF) (frequency)	0	57.90 ± 22.84	3.95	-	<0.05
	1	56.26 ± 19.41			
	≥2	62.13 ± 19.59			

Binary variables (e.g., place of accommodation, the only child) were analyzed using independent-samples *t*-test, with results presented as *t*-value; multinomial variables (e.g., age, education) were analyzed using one-way ANOVA, with results presented as *F*-value. M ± SD represents mean ± standard deviation. *p* < 0.05 was considered statistically significant.

### Descriptive statistics and correlation analysis

Table 3 presents means, standard deviations, and Pearson correlation coefficients for all study variables. Psychological distress (*M* = 58.56, *SD* = 20.91) demonstrated statistically significant and moderately strong positive correlations with fertility stress (*r* = 0.43, *p* < 0.01), rumination (*r* = 0.53, *p* < 0.01), and negative coping styles (*r* = 0.49, *p* < 0.01).

**Table 3:** Correlations among variables.

Variable	Mean ± SD	V1	V2	V3	V4	V5
V1	58.56 ± 20.91	1				
V2	140.53 ± 45.39	0.43**	1			
V3	33.45 ± 14.26	0.53**	0.37**	1		
V4	30.32 ± 9.79	−0.07	−0.11**	−0.05	1	
V5	22.52 ± 7.03	0.49**	0.45**	0.44**	0.08*	1

This table displays the mean and coefficient of variation for each variable, along with the correlation values between variables. Variables included are: V1, Psychological distress; V2, Fertility stress; V3, Rumination; V4, Positive coping styles; V5, Negative coping styles. Significance levels are indicated by **p < 0.01; *p < 0.05.

Fertility stress was positively correlated with both rumination (*r* = 0.37, *p* < 0.01) and negative coping styles (*r* = 0.45, *p* < 0.01), supporting the hypothesized relationships in the mediation model. Rumination was also positively associated with negative coping styles (*r* = 0.44, *p* < 0.01). Notably, positive coping styles showed no significant correlation with psychological distress (*r* = −0.07, *p* > 0.05) in the bivariate analysis. This non-significant association indicates only that no clear relationship between positive coping styles and distress was identified in the current sample; it does not permit conclusions regarding the causal role or relative importance of positive coping styles in the fertility stress–distress pathway. Consistent with our *a priori* theoretical rationale grounded in Response Styles Theory and prior infertility research suggesting that psychological distress is driven primarily by active engagement innegative behaviors rather than by deficits in adaptive styles. Therefore, the subsequent mediation analyses focused exclusively on negative coping styles.

Correlation analysis revealed significant associations among the key variables (Table 3). Given the potential for multicollinearity due to these correlations, formal diagnostics were conducted prior to testing the mediation model.

Multicollinearity diagnostics showed that tolerance values ranged from 0.71 to 0.77, and variance inflation factor (VIF) values ranged from 1.30 to 1.40 (Table 4). All VIF values were well below the commonly accepted threshold of 5, indicating that serious multicollinearity was not present among the predictor variables.

**Table 4:** Multicollinearity diagnostics of predictor variables.

Variable	Tolerance	VIF
Fertility stress	0.77	1.31
Rumination	0.77	1.30
Negative coping styles	0.71	1.40

VIF, Variance Inflation Factor; Tolerance, 1/VIF. All VIF values were substantially below the commonly accepted threshold of 5 and all tolerance values were above 0.10, indicating that serious multicollinearity was not present. These diagnostics support the validity of the regression estimates in the mediation model, though they do not exclude the possibility of modest construct overlap among self-reported variables.

### Chain mediation analysis

#### Regression models for mediating pathways

Before testing the mediation model, we examined the direct effects of fertility stress on the mediators and outcome variable. As shown in Table 5, fertility stress significantly predicted rumination (*b* = 0.11, SE = 0.01, 95% CI [0.09, 0.14], *t* = 9.52, *p* < 0.001).

**Table 5:** Regression analysis on model variables.

Outcome variable	Predictive variable	*R*^2^	*F*	*b*	Ses	*t*	LLCI	ULCI
Psychological distress	Fertility stress	0.39	64.51	0.09***	0.02	5.20	0.05	0.12
	Rumination			0.52***	0.05	9.78	0.42	0.63
	Negative coping styles			0.72***	0.11	6.37	0.50	0.94
Negative coping styles	Fertility stress	0.29	50.06	0.05***	0.01	8.74	0.04	0.06
	Rumination			0.16***	0.02	8.70	0.12	0.19
Rumination	Fertility stress	0.14	24.33	0.11***	0.01	9.52	0.09	0.14

This table presents the regression analysis results of the model variables, with age, employment status, and treatments with *in vitro* fertilization (IVF) as control variables. The table lists the outcome and predictive variable, *R*^2^, *F*-statistic, unstandardized coefficient (*b*), standard error (Ses), *t*-value, and 95% confidence intervals (LLCI and ULCI). Predictors like fertility stress and rumination significantly relate to outcomes like psychological distress and coping styles at the *p* < 0.001 level, marked by ***. LLCI, Lower Level Confidence Interval; ULCI, Upper Level Confidence Interval.

Fertility stress and rumination together significantly predicted negative coping styles (*R*^2^ = 0.29, *F* = 50.06, *p* < 0.001). Specifically, fertility stress directly predicted negative coping styles (*b* = 0.05, SE = 0.01, 95% CI [0.04, 0.06], *t* = 8.74, *p* < 0.001), as did rumination (*b* = 0.16, SE = 0.02, 95% CI [0.12, 0.19], *t* = 8.70, *p* < 0.001).

Finally, fertility stress, rumination, and negative coping styles collectively predicted psychological distress (*R*^2^ = 0.39, *F* = 64.51, *p* < 0.001). In this full model, fertility stress maintained a significant direct effect on psychological distress (*b* = 0.09, SE = 0.02, 95% CI [0.05, 0.12], *t* = 5.20, *p* < 0.001). Rumination (*b* = 0.52, SE = 0.05, 95% CI [0.42, 0.63], *t* = 9.78, *p* < 0.001) and negative coping styles (*b* = 0.72, SE = 0.11, 95% CI [0.50, 0.94], *t* = 6.37, *p* < 0.001) also significantly predicted psychological distress, with negative coping styles showing the strongest predictive effect.

#### Total, direct, and indirect effects

Table 6 presents the decomposition of the total effect of fertility stress on psychological distress into direct and indirect components, with age, employment status, and treatments with *in vitro* fertilization (IVF) as control variables. The bootstrap analysis with 5,000 resamples revealed a significant total effect (*b* = 0.20, SE = 0.02, 95% CI [0.16, 0.23]).

**Table 6:** Mediating effect analysis for rumination and negative coping styles.

Pathway	Effect	SE	Boot LLCI	Boot ULCI	Relative mediating effect
Total effect	0.20	0.02	0.16	0.23	100%
Direct effect	0.09	0.02	0.05	0.12	45%
Total indirect effect	0.11	0.01	0.09	0.13	55%
Ind1	0.06	0.01	0.04	0.08	30%
Ind2	0.04	0.01	0.02	0.05	20%
Ind3	0.01	0.00	0.01	0.02	5%

Mediating effect analysis for rumination and negative coping styles. The table shows the total effect, direct effect, and total indirect effects of fertility stress on psychological distress through rumination and negative coping styles. It includes the effect size, standard error (SE), bias-corrected 95% confidence intervals (BootLLCI and BootULCI), and the relative mediating effect for each pathway. Indirect effect 1: Fertility stress → Rumination → Psychological distress; Indirect effect 2: Fertility stress → Negative coping styles → Psychological distress; Indirect effect 3: Fertility stress → Rumination → Negative coping styles → Psychological distress. Abbreviation: Boot LLCI, Bootstrap Lower Level Confidence Interval; Boot ULCI: Bootstrap Upper Level Confidence Interval.

The direct effect of fertility stress on psychological distress, after accounting for the mediating roles of rumination and negative coping styles, remained significant (*b* = 0.09, SE = 0.02, 95% CI [0.05, 0.12]), accounting for 45% of the total effect.

The total indirect effect through all mediating pathways was *b* = 0.11 (SE = 0.01, 95% CI [0.09, 0.13]), representing 55% of the total effect.

**Figure 1:**
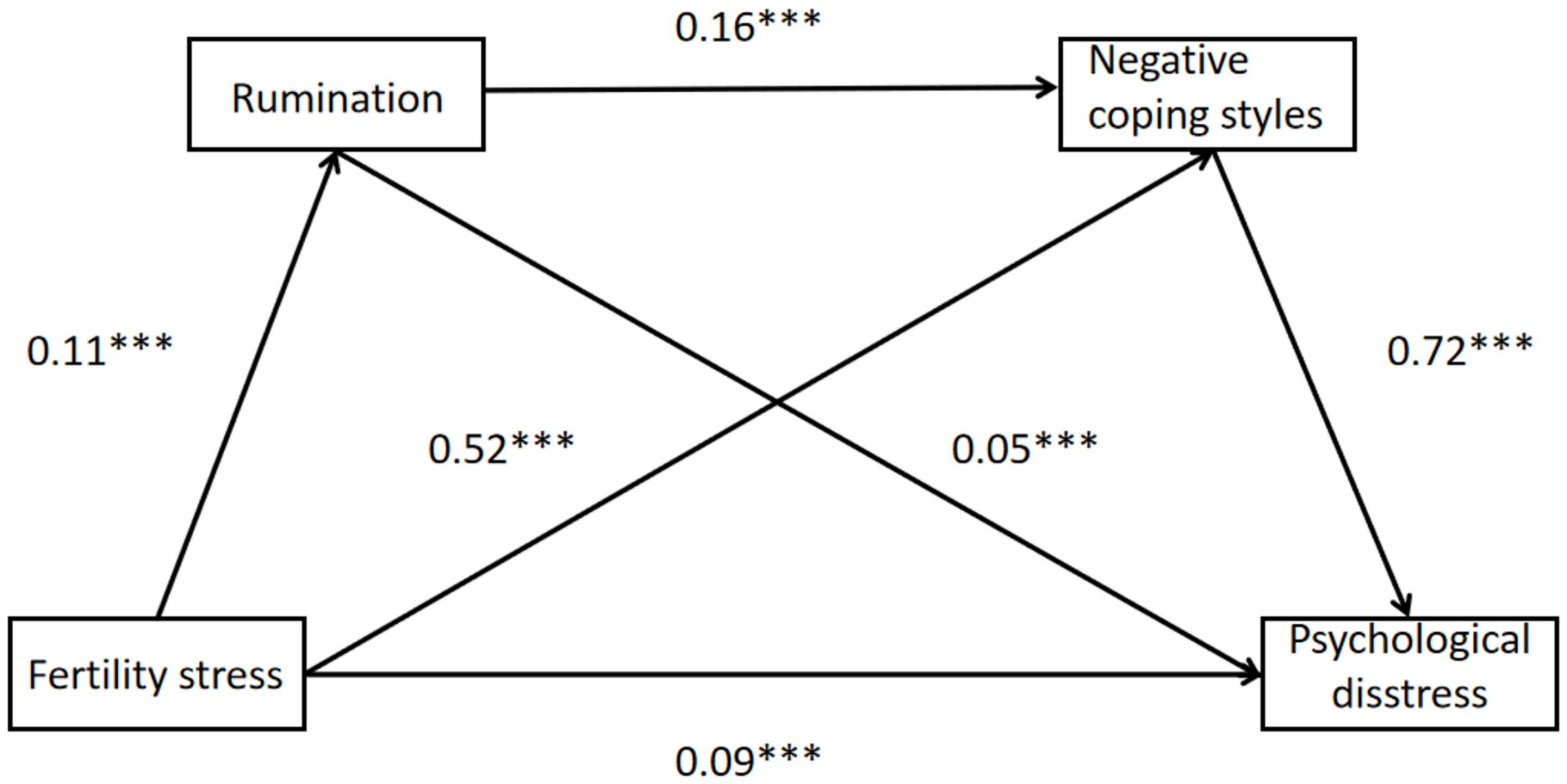
Mediating effects of rumination and negative coping styles on the relationship between fertility stress and psychological distress. All indirect effects are statistically significant as the 95% confidence intervals do not include zero. The unstandardized coefficients are indicating significance at the *p* < 0.001 level, marked by ***.

Three specific indirect pathways were tested, as illustrated in Figure 1, all of which demonstrated statistical significance (95% CIs did not include zero):

Indirect Effect 1 (Fertility stress → Rumination → Psychological distress): *b* = 0.06, SE = 0.01, 95% CI [0.04, 0.08], accounting for 30% of the total effect.

Indirect Effect 2 (Fertility stress → Negative coping styles → Psychological distress): *b* = 0.04, SE = 0.01, 95% CI [0.02, 0.05], accounting for 20% of the total effect.

Indirect Effect 3 (Chain pathway: Fertility stress → Rumination → Negative coping styles → Psychological distress): *b* = 0.01, SE = 0.00, 95% CI [0.01, 0.02], accounting for 5% of the total effect.

## Discussion

Psychological distress, a key indicator of mental health, has gained significant attention in recent years. The relationship between stressors and psychological distress has become a focal point in research. Previous studies have demonstrated that fertility stress positively predicts psychological distress in infertile women (Dong et al., 2022), although the underlying mediating pathways remain unclear. This study examined the relationships among fertility stress, rumination, negative coping styles, and psychological distress in infertile women. Our findings confirmed that fertility stress is directly and positively associated with rumination, psychological distress, and negative coping styles—identifying these as key risk factors. More importantly, we identified both independent and sequential mediating pathways through which fertility stress influences psychological distress outcomes. It should be noted that the cross-sectional design of this study limits the interpretation of the relationships among variables. Although we employed a sequential mediation framework that assumes a directional association (fertility stress → rumination → negative coping styles → psychological distress), the cross-sectional nature of the data prevents us from establishing temporal ordering or verifying causal mechanisms.

### The relationship of fertility stress with psychological distress

Our findings revealed that fertility stress was significantly and positively related to psychological distress, validating Hypothesis 1 and aligned with previous research findings (Facchin et al., 2019) indicating that the psychological distress levels increase with fertility stress. Stress arises when individuals encounter obstacles while pursuing meaningful goals or fail to successfully cope with negative stimuli (Liu et al., 2022) Infertile patients frequently experience fertility stress from multiple sources including expectations, societal pressures, and internal goal-striving failures. For Chinese women in particular, this fertility stress is deeply intertwined with culturally-specific social dynamics that extend beyond those documented in Western samples. Rooted in Confucian ethics that frame childbearing as a core expression of filial piety and family continuity, infertile Chinese women face intense and often relentless pressure from spouses, parents-in-law, and extended family networks to fulfill the reproductive role (Taebi et al., 2021). This pressure is qualitatively distinct from general social expectations: it is embedded in intergenerational family obligation, where childlessness may be experienced not only as a personal loss but as a failure of familial duty and a source of collective shame (Gouni et al., 2022). Compounding this, infertility-related stigma remains pervasive in Chinese society, where childlessness continues to be misattributed to personal moral failing or physical inadequacy, exposing women to social exclusion, gossip, and unsolicited questioning from community networks (Zhang et al., 2021).

Critically, these culturally-specific stressors, family pressure, social stigma, and the internalized obligation to fulfill the maternal role, are not merely background conditions but are likely to act as proximal triggers for the cognitive and behavioral mechanisms identified in this study. Women who internalize culturally-prescribed reproductive expectations may be particularly prone to rumination: the perceived gap between cultural ideals and reproductive reality creates unresolvable cognitive tension that drives repetitive, involuntary self-referential thinking. Similarly, the social imperative to conceal infertility-related distress, stemming from stigma and the desire to maintain family harmony (face-saving), may specifically promote avoidance and denial as dominant coping responses in this population. These cultural mechanisms provide contextual grounding for understanding why rumination and negative coping styles emerged as the primary mediating pathways in this Chinese sample, and suggest that culturally-adapted interventions addressing stigma and family pressure alongside cognitive techniques may be particularly warranted.”

Notably, fertility stress maintained a significant direct effect (*b* = 0.09, representing 45% of the total effect) even after accounting for the mediating pathways identified in this study. This substantial direct effect indicates that fertility stress influences psychological distress through mechanisms beyond the cognitive-emotional pathways examined here, suggesting that other processes (e.g., biological stress responses, practical hardships) merit investigation. In the Chinese cultural context, these unexamined mechanisms may also include the psychological impact of navigating infertility-related social stigma in daily life, the stress of balancing traditional family expectations with personal well-being, and the practical and emotional toll of seeking fertility treatments amid societal and family pressures—all of which warrant further exploration in future research to better understand the full scope of fertility stress’s influence on psychological distress among Chinese infertile women.

### Three mediating pathways: a comprehensive analysis

Our mediation analyses identified three distinct statistical associational pathways through which fertility stress is linked to psychological distress, each representing a different dimension of psychological vulnerability relevant to the observed associations.

#### Independent mediating pathways

First, rumination served as an independent statistical mediator, accounting for 30% of the total association between fertility stress and psychological distress. This finding extends prior research by demonstrating that rumination operates as a discrete cognitive associational pathway in the infertility context: even when the sequential chain through negative coping styles is not activated, rumination alone transmits a meaningful portion of the association between fertility stress and psychological distress (Ando et al., 2020). This is consistent with Response Styles Theory and corroborates evidence from other clinical populations in which rumination is independently associated with emotional distress.

Second, negative coping styles emerged as the largest independent statistical mediating pathway, accounting for 20% of the total association between fertility stress and psychological distress. This finding validates Hypothesis 3 and aligns with research demonstrating an association between infertility-related stress and the adoption of negative coping styles such as avoidance and denial, which are linked to greater distress (Adane et al., 2024). Both cognitive and behavioral factors are independently associated with the vulnerability to distress observed in the current sample. Within the Chinese cultural context, avoidance and denial may be further reinforced by stigma-driven concealment norms: the social imperative to maintain harmony and protect family reputation may discourage women from openly seeking support or expressing distress, thereby entrenching reliance on these negative coping styles.

### The sequential chain pathway: a critical vulnerability mechanism

Beyond these independent effects, we identified a theoretically and clinically relevant sequential chain pathway: fertility stress → rumination → negative coping styles → psychological distress (Hypothesis 4 confirmed). Notably, given the relatively small indirect effect size of this chain pathway (accounting for 5% of the total effect), interpretations of this sequential mechanism should remain cautious.

The sequential ordering of this pathway is theoretically coherent: consistent with Response Styles Theory and the Stress-Cognitive Vulnerability Interaction Model, rumination is associated with depleted cognitive resources, which in turn is linked to reduced capacity for effective coping and greater reliance on negative behavioral responses (Int-Veen et al., 2024). This sequential associational pathway advances existing literature by identifying a specific cognitive-to-behavioral associational sequence in the infertility context, rather than treating rumination and coping as independent stress responses.

This sequential associational pathway contributes to the literature by identifying a specific cognitive-behavioral associational sequence, rather than treating rumination and coping as independent responses to stress. The pathway suggests a pattern of vulnerability amplification: each step in the proposed sequence (fertility stress → rumination → negative coping styles → psychological distress) is linked to a compounded psychological burden. Early intervention targeting rumination could theoretically interrupt this escalation, preventing downstream adoption of negative coping styles — though this possibility requires further empirical validation, given the small effect size of this secondary pathway.

### The non-significance of positive coping styles

An important observation requires mention: positive coping styles showed no significant correlation with psychological distress (*r* = −0.07, *p* > 0.05) in the bivariate analysis. This non-significant association, derived from cross-sectional self-report data, indicates only that no clear relationship between positive coping styles and distress was identified in the current sample, rather than supporting definitive conclusions about the role of positive coping styles in predicting distress or determining intervention priorities.

This pattern is theoretically consistent with our *a priori* focus on negative coping styles: Response Styles Theory and prior empirical work suggest that distress escalation is driven primarily by active negative engagement rather than by deficits in positive styles. However, a non-significant bivariate correlation cannot establish whether positive coping styles is clinically irrelevant to distress in this population, nor can it support comparative conclusions about the relative efficacy of reducing negative coping styles versus building adaptive coping as intervention styles. Whether these theoretical distinctions translate into differential intervention outcomes remains an open empirical question that requires direct testing through experimental or longitudinal designs.

### Clinical implications and intervention targets

Beyond statistical significance, our findings suggest directions for intervention development. Rumination and negative coping styles together account for 55% of fertility stress’s effect on psychological distress. Importantly, we note that these findings suggest potential intervention targets rather than establish proven clinical pathways. Given the cross-sectional design of this study, the following are best understood as preliminary implications warranting testing through randomized controlled trials.

Unlike fertility stress itself (a distal stressor external to the individual), rumination represents an internal cognitive process amenable to evidence-based psychological techniques. Cognitive-behavioral interventions targeting rumination—including cognitive restructuring, metacognitive therapy, and rumination-focused mindfulness—may merit priority in psychological support programs for infertile women.

The sequential pathway (fertility stress → rumination → negative coping styles → psychological distress) suggests potential staged interventions: (1) Early intervention to reduce rumination during the stress→rumination phase, potentially preventing negative coping styles escalation; (2) Concurrent intervention addressing both rumination and established negative coping styles; (3) Risk monitoring using rumination as an early indicator of distress. However, without RCT evidence, specific clinical implementation recommendations cannot be made. Future RCTs testing whether rumination-focused interventions reduce distress in infertile populations are essential before clinical practice changes.

### Methodological considerations

#### Common method Bias assessment

This study relies on self-reported data collected at a single timepoint. While Harman’s single-factor test (27.60% < 40% threshold) suggested limited overt common method bias (Wang et al., 2022), we recognize that method variance may have modestly inflated observed associations. However, the strong theoretical grounding of our hypothesized pathways, the presence of meaningful effect sizes even for smaller pathways (5% chain mediation), and consistency with prior empirical literature suggest that core findings are unlikely to be purely artifacts of measurement method. Nonetheless, future research employing multi-method measurement styles (e.g., physiological indicators, behavioral observation, clinician-rated assessments) with temporal separation of measurement would further strengthen confidence in these relationships.

#### Interpretation of explained variance

The mediation model explained 39% of the variance in psychological distress (R^2^ = 0.39). Formal multicollinearity diagnostics confirmed that VIF values (range: 1.30–1.40) were well below the threshold of 5, indicating that the predictor variables retain sufficient independence and that multicollinearity does not account for this level of explained variance. Nevertheless, a balanced interpretation is warranted: the explained variance likely reflects a combination of (1) theoretically meaningful associations among constructs integrated within a chain mediation pathway, (2) the homogeneous and domain-specific nature of the sample, and (3) potential residual overlap inherent in self-reported constructs collected simultaneously. Readers should therefore interpret the magnitude of explained variance in light of these factors, and future multi-method or longitudinal studies would provide stronger evidence for the robustness of the identified pathways.

### Note on rumination dimensions

While the Event-Related Rumination Inventory (ERRI) measures both intrusive rumination (involuntary, repetitive thinking) and deliberate rumination (intentional attempts at meaning-making) (Seliniotaki et al., 2021), our primary analyses examined rumination as a unitary construct. Both dimensions involve cognitively demanding processing in the infertility context. Future research would benefit from examining whether intrusive and deliberate rumination exert differential protective or risk effects on psychological distress in infertile populations, as these may have distinct intervention implications.

### Limitations and future research

Despite the contributions of this study, several important limitations must be acknowledged.

### Causal inference limitations of cross-sectional design

First, the cross-sectional design prevents empirical verification of the temporal ordering assumed in the chain mediation model. Although Response Styles Theory and the Stress-Cognitive Vulnerability framework provide strong theoretical justification for the hypothesized sequence (fertility stress → rumination → negative coping styles → psychological distress), this pathway remains theoretically inferred rather than empirically verified.

Second, alternative causal pathways cannot be ruled out: (a) reverse associations in which distress may amplify the perception of fertility stress; (b) bidirectional associations in which rumination and coping may mutually reinforce each other; or (c) parallel associational pathways in which fertility stress is independently linked to both rumination and coping. Given the cross-sectional design of this study, temporal ordering and causal inferences cannot be established. Although the present study employed a sequential statistical mediation framework depicting a proposed directional pathway (fertility stress → rumination → negative coping styles → psychological distress), this framework reflects a statistical associational model rather than a verified causal mechanism. Alternative explanations remain highly plausible: for instance, psychological distress may be associated with increased rumination, may be linked to greater use of negative coping strategies, and may also shape perceptions of fertility-related stress. Therefore, all findings should be interpreted as statistical associational mediation rather than causal mediation. These points further highlight the need for caution when interpreting the observed pathways, and longitudinal designs are warranted to clarify potential temporal, reverse, and bidirectional relationships among these variables in future research.

Third, unmeasured confounding variables—including personality traits (trait rumination, neuroticism), contextual factors (social support, stigma, treatment characteristics), and prior life experiences (trauma, mental health history)—could account for the observed associations, creating the appearance of mediation rather than reflecting true causal mechanisms.

To address these limitations, future research should employ: (1) longitudinal or prospective designs measuring variables at multiple timepoints; (2) experience sampling methods (ecological momentary assessment) to capture daily stress dynamics; (3) randomized controlled trials targeting rumination to confirm its causal role; and (4) structural equation modeling with multi-wave longitudinal data to test competing causal models.

### Measurement and content limitations

Fourth, important variables were not measured, including social support, social stigma, treatment characteristics, coping resources (e.g., resilience, self-efficacy), treatment stage, and financial burden. In particular, the absence of direct measures of infertility-related stigma experience, family pressure intensity, and face-saving concerns represents a limitation given the culturally-specific hypotheses advanced in this discussion. Future research should incorporate validated measures of these cultural variables to empirically test whether they moderate the pathways identified in the present model.

Fifth, it should be noted that the present study has a limitation regarding the generalizability of its findings. Specifically, convenience sampling was employed to recruit participants exclusively from the outpatient clinics and inpatient wards of the reproductive medicine departments in three tertiary hospitals. As a result, the sample is predominantly composed of urban women who sought treatment at tertiary medical institutions, which may not be representative of all infertile women. This sampling approach and sample characteristics may limit the generalizability of the study results, as they cannot fully reflect the characteristics and experiences of infertile women from rural areas, those receiving treatment at primary or secondary hospitals, or those with different socioeconomic backgrounds. Therefore, caution should be exercised when generalizing the current findings to a broader population of infertile women.

Sixth, a limitation of the present study relates to the assessment of common method bias (CMB) using Harman’s single-factor test. While this test—wherein all items from the four main scales were entered into an unrotated exploratory factor analysis, with no single factor accounting for more than 40% of the total variance—provided a preliminary check for CMB, it has inherent limitations. This traditional method is simplistic and cannot effectively distinguish between latent method effects and true relationships among variables, nor can it identify complex forms of method bias. As such, the test results should be interpreted cautiously and cannot be considered definitive evidence of the complete absence of CMB. We have therefore adopted a prudent stance when interpreting the observed variable associations, accounting for potential method variance that may not have been fully captured.

Finally, longitudinal or randomized controlled trial evidence confirming that rumination reduction leads to decreased psychological distress would strengthen clinical recommendations for infertility treatment centers.

## Conclusion

This cross-sectional study with 608 Chinese infertile women demonstrates that fertility stress influences psychological distress through direct effects and three indirect pathways: via rumination, via negative coping styles, and sequentially through rumination to negative coping styles. Collectively, these mediators account for 55% of the total effect.

The sequential chain mediation model identifies a theoretically meaningful vulnerability pathway: within Response Styles Theory, rumination precedes negative coping styles because it depletes cognitive resources and impairs problem-solving. While the cross-sectional design prevents empirical verification of this temporal sequence, theoretical rationale suggests rumination-targeted interventions may prevent downstream negative coping styles—though longitudinal research is needed to confirm this causal process.

Clinical Implications (Preliminary): While our findings identify rumination and negative coping styles as factors associated with psychological distress in infertile women, we emphasize that clinical recommendations remain preliminary and contingent on future intervention research. Potential clinical approaches may include: Screening for rumination and negative coping styles patterns as part of comprehensive reproductive health assessment (to identify women at elevated distress risk); considering rumination as a potential intervention target in future treatment development. However, we caution that without randomized controlled trial evidence, specific recommendations regarding intervention type, timing, or intensity cannot be made. The current evidence establishes associations, not causal pathways. Practitioners should await intervention trial findings before implementing rumination-focused therapies as a primary clinical strategy.

Future Research Imperatives: Longitudinal studies and randomized controlled trials are essential to: (1) establish causal directionality and temporal sequences; (2) test whether interventions reducing rumination effectively decrease psychological distress; (3) compare effectiveness of rumination-focused versus other approaches (e.g., coping enhancement, behavioral activation); (4) determine generalizability across cultural and clinical contexts; and (5) incorporate key psychosocial and contextual variables—including perceived social support, infertility-related stigma, marital satisfaction, treatment stage, and financial burden—into comprehensive models to expand the explanatory scope of research on infertility-related psychological distress and provide a more holistic understanding of the complex factors influencing the associations among fertility stress, rumination, negative coping styles, and psychological distress.

## Data Availability

The raw data supporting the conclusions of this article will be made available by the authors, without undue reservation.
